# Right‐Sided Infective Endocarditis Associated With a Hemodialysis Catheter

**DOI:** 10.1002/ccr3.73609

**Published:** 2026-10-01

**Authors:** Said Abdirahman Ahmed

**Affiliations:** ^1^ Department of Cardiology Mogadishu Somali Turkish Training and Research Hospital Mogadishu Somalia

**Keywords:** echocardiography, hemodialysis catheter, right‐sided infective endocarditis, septic pulmonary emboli, tricuspid valve vegetation

## Abstract

In hemodialysis patients with fever and septic pulmonary emboli, mobile right‐sided vegetations should prompt evaluation for catheter‐associated infective endocarditis and early catheter removal.

## 1 Case Presentation

A 45‐year‐old man on hemodialysis via a tunneled catheter presented with fever, chest pain, and dyspnea. CT showed septic pulmonary emboli. TTE demonstrated two mobile tricuspid vegetations (0.61 × 0.75 cm and 1.48 × 1.75 cm) with moderate regurgitation (Figure 1A–C; Video S1). Cultures were obtained before daptomycin 320 mg and meropenem 1 g daily; cultures remained negative after prior antibiotics. The catheter was removed. TEE and follow‐up were precluded by self‐discharge [1, 2].

**FIGURE 1:**
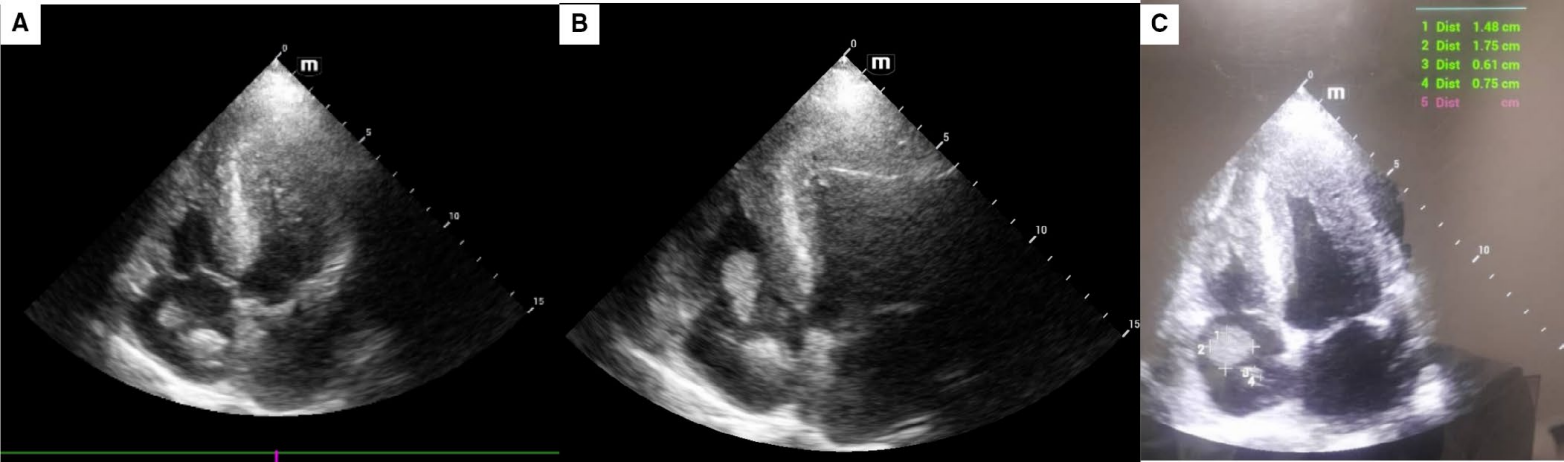
Apical four‐chamber transthoracic echocardiographic images demonstrating two highly mobile echogenic vegetations involving the tricuspid valve region. (A) Shows the vegetations within the right atrium adjacent to the tricuspid valve annulus. (B) Shows prolapse through the tricuspid valve into the right ventricle. (C) Demonstrates the two discrete vegetations, measuring 0.61 × 0.75 cm and 1.48 × 1.75 cm, respectively.

## Author Contributions

**Said Abdirahman Ahmed:** conceptualization, writing – review and editing, resources, writing – original draft, data curation.

## Funding

The author has nothing to report.

## Consent

Written informed consent was obtained from the patient for publication of this image report and Video S1.

## Conflicts of Interest

The author declares no conflicts of interest.

## Supporting information


**Video S1:** Apical four‐chamber transthoracic echocardiographic cine loop demonstrating highly mobile tricuspid vegetations with prolapse across the tricuspid valve during the cardiac cycle.

## Data Availability

The data that support the findings of this study are available on request from the corresponding author. The data are not publicly available due to privacy or ethical restrictions.
